# Preparation of Dry Extract of *Mikania glomerata* Sprengel (Guaco) and Determination of Its Coumarin Levels by Spectrophotometry and HPLC-UV

**DOI:** 10.3390/molecules170910344

**Published:** 2012-08-29

**Authors:** Luciana Soares e Silva, Luciane Santos da Silva, Larissa Brumano, Paulo César Stringheta, Miriam Aparecida de Oliveira Pinto, Leticia Oliveira Moreira Dias, Camila de Sá Martins Muller, Elita Scio, Rodrigo Luiz Fabri, Helena C. Castro, Maria da Penha Henriques do Amaral

**Affiliations:** 1 Faculty of Pharmacy, Federal University of Juiz de Fora, Juiz de Fora 36036-900, MG, Brazil; Email: lucianesantos.far@gmail.com (L.S.S.); larissabrumano@gmail.com (L.B.); miriamaop@yahoo.com.br (M.A.O.P.); leticiadias.farma@gmail.com (L.O.M.D.); camilamuller@ecofarmajr.com.br (C.S.M.M.); penhaufjf@yahoo.com.br (M.P.H.A.); 2 Federal University of Viçosa, Viçosa 36570-900, MG, Brazil; Email: pstringheta@yahoo.com.br; 3 Institute of Biological Sciences, Federal University of Juiz de Fora, Juiz de Fora 36036-900, MG Brazil; Email: elita.scio@ufjf.edu.br (E.S.); rodrigolfabri@yahoo.com.br (R.L.F.); 4 Institute of Biology, Fluminense Federal University, Niterói 24220-900, RJ, Brazil; Email: hcastrorangel@yahoo.com.br

**Keywords:** pharmacotechnique, freeze-dryer, lyophilization, coumarins

## Abstract

Guaco (*Mikania glomerata *Sprengel) syrup is one of the most popular herbal medicines used to treat the symptoms of asthmatic bronchitis, cough and hoarseness. The coumarin 2*H*-1-benzopyran-2-one, is one of the major constituents of Guaco and contributes to its pharmacological effects. The pharmaceutical capsule form of dry extract of Guaco is recommended by the Brazilian Program of Medicinal Plants and Herbal Medicines and used in primary health care. In order to identify a new protocol to obtain the raw material for Guaco capsule production we evaluated two methods, including a freeze-drying process (lyophilization) and the spray-dryer technique, as well as the use of two adjuvants, Maltodextrins and Aerosil^®^, in different concentrations. The coumarin levels of the dried extracts were analyzed by UV-spectrophotometry and HPLC-UV/DAD. The adjuvant Aerosil^®^ 8% showed better dry powder physical appearance. Lyophilization was observed to be the best process to obtain the dry extract of Guaco based on the measured coumarin levels.

## 1. Introduction

*Mikania glomerata* Sprengel, popularly known as Guaco, is a plant from the Asteraceae family with medicinal properties. Guaco is used as an effective natural bronchodilator, expectorant and cough suppressant in Brazilian herbal medicine. It is employed for different upper respiratory problems including bronchitis, pleurisy, coughs, asthma, colds and flu. Guaco syrup produced with the alcoholic fluid extract is the pharmaceutical form commonly used in the therapeutic of upper airways diseases [1,2,3].

*M. glomerata *is cited in the Normative Instruction Number 5 of the Brazilian National Health Surveillance Agency (ANVISA), which publishes the “List of Phytotherapeutic Drugs of Simplified Registration”. According to this list, the herbal medicines obtained from the species described therein (n = 71) do not require safety and efficacy validation since their therapeutic and safe use are widely reported in several databases including COCHRANE, PUBMED, BVS-Medline, BVS-Lilacs, and Coordination of Improvement of Higher Education Personnel (CAPES). Besides syrup, Guaco capsules is another pharmaceutical form produced from dry extract that appears in the list of phytotherapeutic drugs of interest of the Unified Health System (SUS) of Brazil [4].

Phytochemical studies of *M. glomerata *showed the presence of ent-caur-16(17)-*en*-19-oic, *ent*-15-β-isobutyloxycaur-16(17)-*en*-19-oic, *ent*-15-β-benzoioxycaur-16(17)-en-19-oic, *ent*-15-β-hydroxy-caur-16(17)-*en*-19-oic, *ent*-17-hydroxycaur-15(16)-*en*-19-oic and *o*-hydroxycinnamic acids, besides stigmasterol, friedelin, β-sitosterol, lupeol and coumarin [5,6,7].

Coumarin, 2*H*-1-benzopyran-2-one, is one of the major chemical constituents of *M. glomerata*, and apparently is involved in the bronchodilator profile of this plant by relaxing the lung smooth muscle [8]. Other tests have also indicated bronchodilating activity, while coumarin was shown to be responsible for about 50 to 60% of this activity in relation to the total activity of the crude extract [9]. 

Natural products with a 2*H*-1-benzopyran-2-one nucleus are widespread in the plant kingdom. Several structural modifications of the benzopyran-2-one nucleus are present in Nature, but by far, simple coumarins characterized by the lack of additional fused ring systems are the most common [10]. 

Coumarins with a 2*H*-1-benzopyran-2-one nucleus, and furocoumarins (psoralens, 7*H*-furo[3,2-g [1]benzopyran-7-one]) are biologically active compounds with anti-convulsant, anti-tumour, anti-inflammatory and anti-viral profiles. Furthermore, coumarins have been widely used as flavoring compounds due to their sweet aromatic odour [11].

Freeze-drying, also known as lyophilization, is widely used for producing pharmaceuticals as it improves labile drug stability during long storage periods (e.g., protein drugs). Freeze-dried formulations not only have the advantage of better stability, but also provide easy handling (shipping and storage) [12]. In the case of Guaco alcoholic fluid extract, prior alcohol evaporation is necessary when using lyophilization. 

Besides the lyophilization process, spray-drying can be used to obtain Guaco dry extract. Spray-drying is defined as the transformation of liquid state feed into a dried particulate form. This is achieved by atomizing the fluid into a drying chamber, where the liquid droplets are passed through a hot-air stream. Thus, this technique is suitable for heat-sensitive products. This drying process is widely used in the large-scale industry to produce instant coffee, milk and fruit juice powders, as well as for the encapsulation of active components [13]. In contrast to lyophilization, the removal of the alcoholic portion is not required in the technique of atomization in spray-dryer.

In this work the use of two adjuvants was evaluated in both the lyophilization and spray-drying processes for obtaining Guaco dry extract. The purpose was to identify the best method that provides the highest coumarin recovery level to generate the raw material for the production of the herbal medicine in solid form.

## 2. Results and Discussion

In a general context, the interest in the use of plant extracts for medicinal purposes has increased in the recent years. In Brazil there are 512 registered phytotherapeutic drugs, of which more than 70% are presented as solid pharmaceutical capsule forms [4]. In general, the raw material for these products consists by dry extracts that present several advantages including greater chemical, physical-chemical and microbiological stabilities, easier standardization, higher concentration of active compounds and higher capacity of transformation in different types of solid pharmaceutical forms [14].

In that context, the selection and use of adjuvants are fundamental steps when using spray drying and lyophilization processes for producing solid pharmaceutical forms. This is especially important for drying plants derived extracts since these adjuvants determine the product stability and quality and also affect the final bioavailability [14].

Aerosil^®^ is a colloidal silicon dioxide widely used as an adjuvant due to its high specific surface and high adsorbent power. Aerosil^®^ presents excellent results when obtaining dried products through atomization from different vegetable species extractive solutions [14].

In drying processes, it is necessary that the final product present reduced internal moisture to avoid microorganism contamination and allow long preservation periods (shelf life) [15,16]. High molecular weight drying agents such as maltodextrin are commonly used to achieve a successful drying in case of sticky products [17]. Some additives such as starch, arabic gum, and maltodextrins are used as support materials to increase the Tg of the products during the spray-drying. Tg is the constant that measures sugar of low glass transition temperature (saccharose, glucose and fructose). The higher the Tg, the higher the presence of sugar. They may either remain as the syrup or stick to the drier chamber walls during drying [18]. 

In this work we evaluated two adjuvants, Aerosil^®^ and Maltodextrin, in preparing Guaco dry extracts using the spray drying and lyophilization processes to identify the best process for producing a dry extract with a high coumarin level. 

The residual humidity for solid herbal medicines should remain between 8% and 14% [19]. After preparing the four Guaco dry extracts using the adjuvants (8 and 10%) with the two different methods we noticed that the humidity values were within the national pharmacopeic specifications. 

The residual humidity data obtained herein when using maltodextrin, was lower than that obtained using Aerosil^®^ (Table 1).

**Table 1:** Residual humidity (%) of the atomized and lyophilized samples using Aerosil^®^ or maltodextrin as adjuvant.

Adjuvants	Samples Residual Humidity (%)	
	Lyophilized	Atomized
Aerosil^®^ 8%	6.40	6.90
Aerosil^®^ 10%	4.69	5.98
Maltodextrin 8%	2.14	5.18
Maltodextrin 10%	1.86	4.68

The influence of the concentration of colloidal silicon dioxide was also analyzed in the drying of extractive solutions of *Maytenus ilicifolia* Martius ex Reissek. The addition of this adjuvant between 10% and 20% in the extraction solution caused a significant reduction in the hygroscopicity of the dried products [14,17].

**Figure 1:**
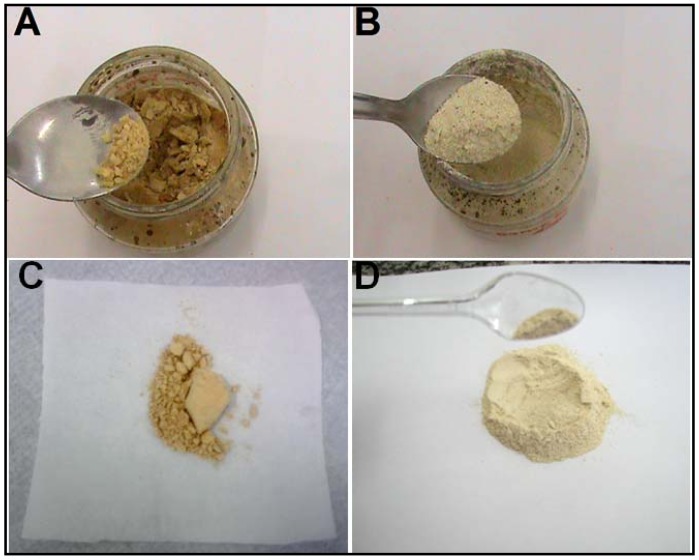
Guaco lyophilized (**A** and **B**) and atomized (**C** and **D**) extracts using maltodextrin (**A** and **C**) or Aerosil^®^ (**B** and **D**) as adjuvant, respectively.

The dried extracts of Guaco obtained using the freeze-drying process (lyophilization) and atomization in a spray-dryer with Aerosil^®^ and maltodextrin (8% and 10%) presented the appearance of a light beige homogeneous dry powder that is considered of good appearance for the development of the solid pharmaceutical capsule form. However, the difference between the two products was that the dry extract obtained with maltodextrin would have to be subjected to soft grinding to obtain a homogenous powder (Figure 1), so from the pharmacotechnical point of view, the use of Aerosil^®^ as adjuvant showed better dry powder physical appearance and density with improved technological features (Figure 1). 

With respect to the mass, the drying processes performed using Aerosil^®^ (8% and 10%) as adjuvant presented a higher Guaco dry extract mass than that obtained with maltodextrin at the same concentration (Table 2). 

**Table 2:** Guaco dry extract Mass (g), coumarin level (mg/g of dry extract) of the lyophilized and atomized dried extracts samples using Aerosil^®^ 8% and 10% or maltodextrin 8% as adjuvants, and the coumarin recovery/yield percentage (%) in each case was in accordance to the calculations obtained from the Equations 1 and 2.

Processes	Adjuvants	Guaco dry extract Mass (g)	Level of Coumarin in dry extract (mg/g)	Yield/recovery of Coumarin (%)
**Lyophilization**	Aerosil^®^ 8%	58.80	47.91	73.32
	Aerosil^®^10%	60.67	50.79	84.54
	Maltodextrin 8%	19.32	48.46	25.68
	Maltodextrin 10%	29.88	50.41	41.32
**Atomization**	Aerosil^®^ 8%	36.00	35.43	34.96
	Aerosil^®^ 10%	49.80	40.78	55.71
	Maltodextrin 8%	20.35	30.08	21.26
	Maltodextrin 10%	21.47	44.58	26.26

**Figure 2:**
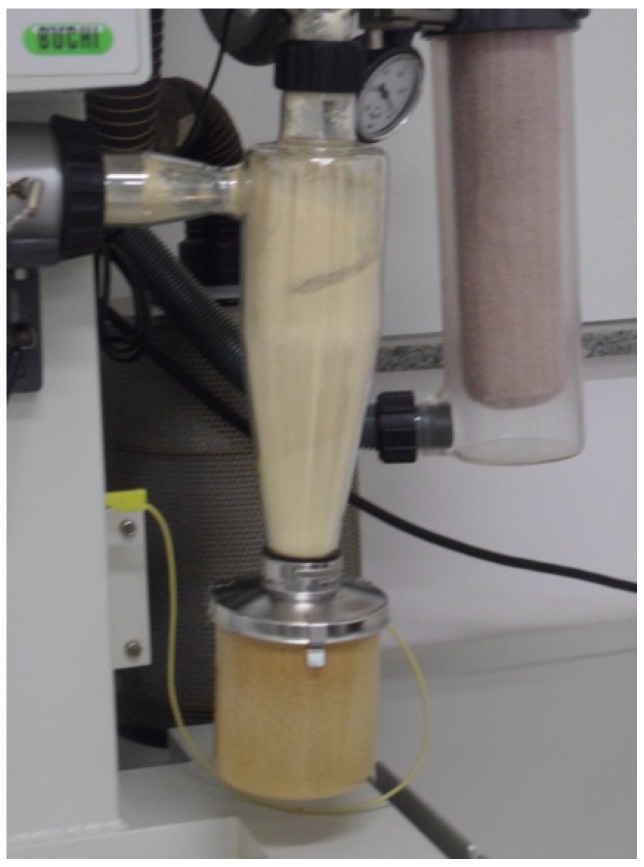
Adhesion of the product in the solid-gas separator and collection system of the spray dryer equipment (Model Büchi Mini Spray Dryer B-191, Büchi Labortechnik AG, Flawil, Switzerland).

Due to the adhesion from powder in the compartments of the drying equipment, a mass loss was observed in the atomization dried product obtained from the spray dryer process compared with lyophilization (Figure 2). Importantly, the use of Aerosil^®^ led to a lower material adhesion in the recipients during the preparation process, which reduced the losses compared to the maltodextrin product. 

The main techniques for the determination of coumarins in *Mikania glomerata* Sprengel and its derivatives are high efficiency liquid chromatography (HPLC) and gas chromatography (GC) [9]. However, due to the nature and position of the chemical substituents, coumarins present a specific ultraviolet spectrum, which allows their identification and development of new spectrophotometric analytical techniques [3,11].

Coumarin levels, obtained by the method of UV-spectrophotometry, of solids products using the adjuvants with 8% in both processes are shown in Table 2. The level of coumarin was also evaluated in the Guaco fluid extract by UV-spectrophotometry [3,20] and the validated technique using HPLC-UV described by Celeghini *et al.* [9], before being subjected to drying and with no adjuvant. Values of 12.15 mg of coumarin per mL of extract by UV-spectrophotometry and 8.8 mg/mL by HPLC-UV were found.

Since no significant difference was observed in the process of preparing Guaco dry extracts when using adjuvants at 8 or 10%, we selected the lower concentration (8%) for the subsequent studies because the smaller amount of excipient would result in better presentation in the capsule. 

The results presented in the Table 3 confirmed that the process of obtaining a guaco dry extract by lyophilization presented a higher level of coumarin as compared to the value found in the product obtained by atomization. Despite the fact that findings of the HPLC method are lower than the UV-spectrophotometry (Table 2), one should take into account that by HPLC, were measured only coumarin 2*H*-1-benzopyran-2-one, while the UV-spectrophotometry identifies the marker 2*H*-1-benzopyran-2-one and also other coumarins present in the composition of the dry and fluid extracts. 

**Table 3:** Coumarin (2*H*-1-benzopyran-2-one) levels (mg/g) analyzed by HPLC-UV method and the yield (%) found in the dried extracts processed by lyophilization and spray dried using Aerosil 8% as adjuvant.

Processes	Level of Coumarin (mg/g)	Yield (%)
Lyophilization	17.7	37.41
Spray-dried	8.12	11.07

According to Araújo, coumarin is a solid and white crystalline material with a melting temperature in the range of 69 to 73 °C. Thus the coumarin content decrease observed for Guaco dried extract from atomization in spray-dryer (Table 2) can be due to the high temperature reached during the atomization process, of about 80 °C [21,22,23].

In this study, for carrying out the spray drying process we used four 300 mL samples under the following conditions: inlet medium temperature of 80 °C, average temperature of the process of 170 °C and feed flow of 4.0 mL/ min. Prior to carrying out the process of atomization, a suspension of fluid hydroalcoholic extract was prepared through additions, separately for each sample, of 8% and 10% of maltodextrin and Aerosil^®^.

The spray-drying process consists of three fundamental steps. In the first phase the fluid is dispersed as droplets, producing a large surface area. In the second, their contact with a stream of heated air with heat transfer occurs. In the third step, there is solvent evaporation and formation of the solid particles [18].

Freeze-drying is considered as one of the best methods to maintain the quality attributes of the materials submitted to drying processes since the combination of the absence of liquid water and low temperature stop most reaction degradations. That is why this process is sometimes used as a reference method to compare drying experiments [24,25]. Cortes *et al.* reported some reasons for the importance of freeze-drying process in the development of functional food, which include: the increase of the product shelf-life, the reduction not only of chemical degradation during storage, but also the products moisture to a level near to that of bonded bonded, which improves the products’ crunchiness characteristics [26].

According to Fellows, the performance of each process is calculated by knowing the amount/concentration of the material of interest, in this case coumarin, before and after the exposure to the drying process [27]. Following these precepts, the first mathematical relationship was obtained: the initial concentration of coumarin (ic), found in the fluid extract of Guaco is multiplied with the initial volume (iv) of that extract before drying in order to obtain the initial total amount of coumarin (iTc) [Equation (1)]:


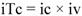
(1)

After the drying process, the second mathematical relationship was obtained: the level of final coumarin of the dried product (fc) dosage is multiplied by the final mass of extract produced (fm) (Table 2). Thus the final overall level of processed coumarin (fTc) is obtained, referring to Equation (2):


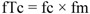
(2)

The percentage relationship (%) between the two values found through the Equations (1) and (2) is established. The initial total amount of coumarin is equivalent to 100% and it is obtained through the Equation (1). The higher the ratio between the initial and the final coumarin levels, the better the process yield. 

## 3. Experimental

### 3.1. General

#### 3.1.1. Plant Material

The Guaco leaves were from the Medical Garden of the Faculty of Pharmacy of the Universidade Federal de Juiz de Fora (UFJF). After harvesting, the leaves were selected and dried in an oven with air circulation under a controlled temperature of 35 °C. A voucher specimen was deposited in the Herbarium Leopoldo Krieger of the UFJF under number 42054.

#### 3.1.2. Hydroalcoholic Fluid Extract Production

The hydroalcoholic fluid extract production from the plant *Mikania glomerata* Sprengel was performed through maceration and percolation, according to the Brazilian Pharmacopoeia 1st edition [28]. The dried leaves were crushed in the machine of ring type and a hammer mill capacity of 3,000 rpm with a 20 mesh sieve (Junqueira-model 10 JC6, Juiz de Fora, Brazil). The 70/30 ethanol/water was the extraction liquid used and the mass/volume ratio was of 1:1.

#### 3.1.3. Preparation of Dried Extracts

We used Guaco hydroalcoholic extract to prepare the formulations, submitting it to lyophilization or to atomization in a spray-dryer adding separately the Aerosil^®^ and Maltodextrin excipients in concentrations that ranged from 8% to 10%. The Terroni Lyophilizer LS 3000 (São Carlos, Brazil) was used for lyophilization process whereas a Büchi Mini Spray Dryer Model B-191 was used for the atomization step of the spray dryer process. 

### 3.2. Coumarin—Method of Analysis

Two analytical methods were used for the determination of coumarin: UV-spectrophotometry and HPLC-UV/DAD analysis.

#### 3.2.1. UV-Spectrophotometry

UV-spectrophotometry (Biochoron Libra S12, Cambridge, UK) at 275 nm was used to prepare a calibration curve using a standard of coumarin 2*H*-1-benzopyran-2-one from Fluka (Sigma-Aldrich, São Paulo, Brazil). The solvent was methanol/water in the ratio 80:20. For the calibration curve determination, seven coumarin standard dilutions were prepared from the stock solution of 480 μg/mL. Thus, a calibration curve was determined with concentrations, in triplicate, of 4.80 µg, 5.76 µg, 6.72 µg, 7.68 µg, 8.64 μg, 9.60 µg and 10.56 µg, yielding the line equation Y = 0.0771X − 0.03 and R^2^ = 0.9961.

To determine the level of coumarin of the dried extracts, a stock solution of 480 µg/mL was prepared by diluting 24 mg of dry extract in 50 mL solution methanol/water (80:20). A second dilution was performed by withdrawing an aliquot of 8 mL of the stock solution in 25 mL of solution methanol/water (80:20) yielding a theoretical final concentration of 153.6 µg/mL. Readings were taken in the spectrophotometer at a wavelength of 275 nm. 

The initial coumarin value (before processing) was determined by analyzing the absorbance of an aliquot of 0.8 mL of Guaco fluid extract diluted into 100 mL of methanol/water (80:20) solution in a spectrophotometer at 275 nm. The value obtained was used in the line equation revealing the level of coumarin present in the fluid extract. 

#### 3.2.2. HPLC Analysis

A modular Agilent Technologic 1200 Series system (Barueri, Brazil) comprised of a LC-10AD pump, a CTO-10A column oven, a SPD-10A UV-DAD detector. A LC-18 column (250 mm × 4 mm i.d. × 5 mm) from Zorbax SB-18 (Barueri, Brazil) was employed, at 25 °C. Separations were done in the isocratic mode, using acetonitrile:water (40:60; v/v) at a flow rate of 1 mL/min; with an injection volume (“loop”) of 20 µL; UV detection was at 274 nm.

### 3.3. Quantitative Analysis

Determination of the content of the coumarin in plant material was performed by the external standard method, using coumarin (2*H*-1-benzopyran-2-one) from Fluka (Sigma-Aldrich) as standard. Stock solutions of 0.5, 1, 10, 20, 40, 60, 80 and 100 µg/mL were utilized. Each determination was carried out in triplicate [9]. 

### 3.4. Determination of Humidity

To determine the humidity content used a Moisture Determinator IR, Mars, Series ID 1.8, ID 50 model, at the Laboratory of Pharmacology, Faculty of Pharmacy, Federal University of Juiz de Fora-MG. The evaluation was performed with extracts of dry Guaco obtained from the spray and freeze drying, containing the adjuvants Aerosil® and maltodextrin in the proportions mentioned in this study.The equipment was adjusted to a maximum temperature of 108 °C and shutdown after stabilizing the sample weight for 1 min. About 1.0 g of each of the material obtained through dehydration techniques described herein was used [29].

## 4. Conclusions

The drying processes analysis data of Guaco hydroalcoholic extract showed that lyophilization produced a dried product with higher coumarin content than that obtained from the spray-dryer. Among the two excipients used, the colloidal silicon dioxide, Aerosil^®^, produced a final product with better dry powder appearance and density with improved technological characteristics, in addition to a lower adhesion in containers during the process, reducing the mass loss percentage.

The dry extract of Guaco obtained by the freeze-drying process was considered adequate as raw material for the production of the solid pharmaceutical capsule form. The UV-spectrophotometry method not only quantified the reference substance, but all coumarins present in Guaco, while the HPLC technique was specific for 2*H*-1-benzopyran-2-one as chemical marker of this species. However the UV-spectrophotometry method has some advantages compared to the HPLC-UV such as reduced operating cost and time of analysis, it is a simple technique with less environmental impact, since the reagent used in the HPLC-UV method, acetonitrile, is teratogenic with a toxic profile by several routes of absorption.
